# Multi-functional nanogel with cascade catalytic performance for treatment of diabetic oral mucosa ulcer

**DOI:** 10.3389/fbioe.2023.1194398

**Published:** 2023-05-23

**Authors:** Yanfen Zheng, Zhiguo Li, Chuyi Liu, Xiaotong Fan, Zheng Luo, Zibiao Li, Yun-Long Wu

**Affiliations:** ^1^ Xiamen Key Laboratory of Stomatological Disease Diagnosis and Treatment, Stomatological Hospital of Xiamen Medical College, Xiamen, Fujian, China; ^2^ China and Fujian College Engineering Research Center for Dental Biomaterials, Xiamen, China; ^3^ Fujian Provincial Key Laboratory of Innovative Drug Target Research, State Key Laboratory of Cellular Stress Biology, School of Pharmaceutical Sciences, Xiamen University, Xiamen, China; ^4^ Institute of Sustainability for Chemicals, Energy and Environment (ISCE2), Agency for Science, Technology and Research (A*STAR), Singapore, Singapore; ^5^ Institute of Materials Research and Engineering (IMRE), Agency for Science, Technology and Research (A*STAR), Singapore, Singapore; ^6^ Department of Materials Science and Engineering, National University of Singapore, Singapore, Singapore

**Keywords:** nanogel, biomaterials, mucosa ulceration, nanoenzyme, wound healing

## Abstract

**Introduction:** Diabetic oral mucosa ulcers face challenges of hypoxia, hyperglycemia and high oxidative stress, which result in delayed healing process. Oxygen is regarded as an important substance in cell proliferation, differentiation and migration, which is beneficial to ulcer recovery.

**Methods:** This study developed a multi-functional GOx-CAT nanogel (GCN) system for the treatment of diabetic oral mucosa ulcers. The catalytic activity, ROS scavenge and oxygen supply ability of GCN was validated. The therapeutic effect of GCN was verified in the diabetic gingival ulcer model.

**Results:** The results showed that the nanoscale GCN was capable of significantly eliminating intracellular ROS, increasing intracellular oxygen concentration and accelerating cell migration of human gingival fibroblasts, which could promote diabetic oral gingival ulcer healing *in vivo* by alleviating inflammation and promoting angiogenesis.

**Discussion:** This multifunctional GCN with ROS depletion, continuous oxygen supply and good biocompatibility, which might provide a novel therapeutic strategy for effective treatment of diabetic oral mucosa ulcers.

## 1 Introduction

The incidence rate of diabetes has been on the rise in the past decade and chronic diabetic wounds are becoming a serious threat to public health (Gregg et al., 2018; Kazemian et al., 2019; Xu et al., 2023). As a diabetic wound type, oral mucosa ulcer is an ulcerative injury occurring in the oral mucosa, which can affect speech, diet, and spirit (ShipMerritt and Stanley, 1962; Pedersen, 1989). Furthermore, oral mucosal lesions are often diagnosed in diabetic patients with a series of alterations occurring such as gingivitis, periodontitis, lichen planus, and recurrent aphthous ulceration (Tsai et al., 2002; Ship, 2003; Vernillo, 2003; Taylor and Borgnakke, 2008; Luo et al., 2020). The major problems of diabetic oral ulcer include hyperglycemia, persistent inflammation, and hypoxia. These complicated physiological milieus hinder oral ulcers from efficiently healing and make them vulnerable to pathogenic bacteria (Falanga, 2005; Shah et al., 2019; Luo et al., 2021a; Liu et al., 2021; Liu et al., 2022).

Currently, topical drugs commonly used in the clinical treatment of oral ulcers include antibiotics, analgesics, and hormone drugs, whose long-term use might cause adverse reactions (Woo and Sonis, 1996). In recent years, a variety of oral patches, including hydrogels, creams, and nanofibers, have been widely developed to deliver antibiotics, glucocorticoids, and so on (Tort and Acarturk, 2016; Logan et al., 2020; Zhang et al., 2021; Zhou et al., 2022; Liu et al., 2023). However, these preparations have problems such as inconvenience in use, strong side effects, or short duration of drug action (Toma et al., 2021). Therefore, safer and more effective multi-functional strategies for oral mucosal healing are urgently required.

Recently, enzymes and nanozymes have shown great prospects in wound healing (Wu et al., 2019; Wang et al., 2020; Zhou et al., 2020; Peng et al., 2021; Sang et al., 2021; Wang et al., 2023; Zhang et al., 2023). A variety of wound dressings containing enzyme or enzyme-like abilities are designed to catalyze endogenous H_2_O_2_, generating O_2_ or OH to improve the diabetic wound environment. Oxygen has been reported to be essential for wound healing because it can promote cell migration, angiogenesis, and other molecular events relate to wound repair processes (Liu et al., 2019; Chen et al., 2020; Zhang et al., 2020; Li et al., 2022a; Li et al., 2022b). However, the sustainability of oxygen in these strategies faces significant obstacles. Furthermore, the complicated physiological environment of high-glucose exposure, high ROS, and hypoxia impairs angiogenesis and oral ulcer healing (Thangarajah et al., 2009). Therefore, a strategy to simultaneously regulating blood glucose, reducing ROS, and continuously delivering oxygen might be effective to accelerate ulcer treatment.

The glucose oxidase (GOx) and catalase (CAT) cascade systems have been extensively studied in the treatment of diabetic ulcers due to the presence of high glucose and high ROS (endogenous H_2_O_2_) (Wang et al., 2021; Li et al., 2022c; Du et al., 2022; Yu et al., 2022). For example, Lei, *et al.* developed a supramolecular cascade reactor with chitosan, sulfobutylether-β-cyclodextrin, Fe^2+^, and GOx. The catalytic oxidation of glucose by GOx in a supramolecular cascade reactor provided H_2_O_2_ for the Fe^2+^-mediated Fenton reaction, which finally generated OH for wound antibacteria (Chen et al., 2022). Similarly, Li et al. (2021) prepared an ionic covalent organic framework–based nanozyme that included Fe^2+^ and GOx to convert glucose and H_2_O_2_ to OH. Li et al. (2022c) designed defect-rich molybdenum disulfide nanosheets loaded with bovine serum albumin–modified gold nanoparticle (MoS_2_@Au@BSA NSs) heterostructures. The GOx-like Au nanoparticles converted glucose into H_2_O_2_, which was transformed intoOH and O_2_ by MoS_2_@Au@BSA. Although these nanozyme systems effectively treat wound healing, their catalytic effectiveness, sustained oxygen supply, and biosafety are of concern because most of these systems use metal or metal oxide enzyme–like compounds. In addition, it is difficult to control the product of H_2_O_2_ conversion to OH or O_2_. Excessive hydroxyl radicals can exacerbate wound inflammation. Therefore, it is crucial to enhance the sustainability of the catalytic reaction and reduce unnecessary products while maintaining the high efficiency of the enzyme. Natural enzyme has high catalytic efficiency and specificity, which makes it popular in wound healing treatment (Li et al., 2022a). As a 3D nanoparticle, nanogels have high colloidal stability, water content, load capacity, and excellent biocompatibility, which make it widely used in the biomedical field (Chacko et al., 2012; Ahmed et al., 2020).

**SCHEME 1:**
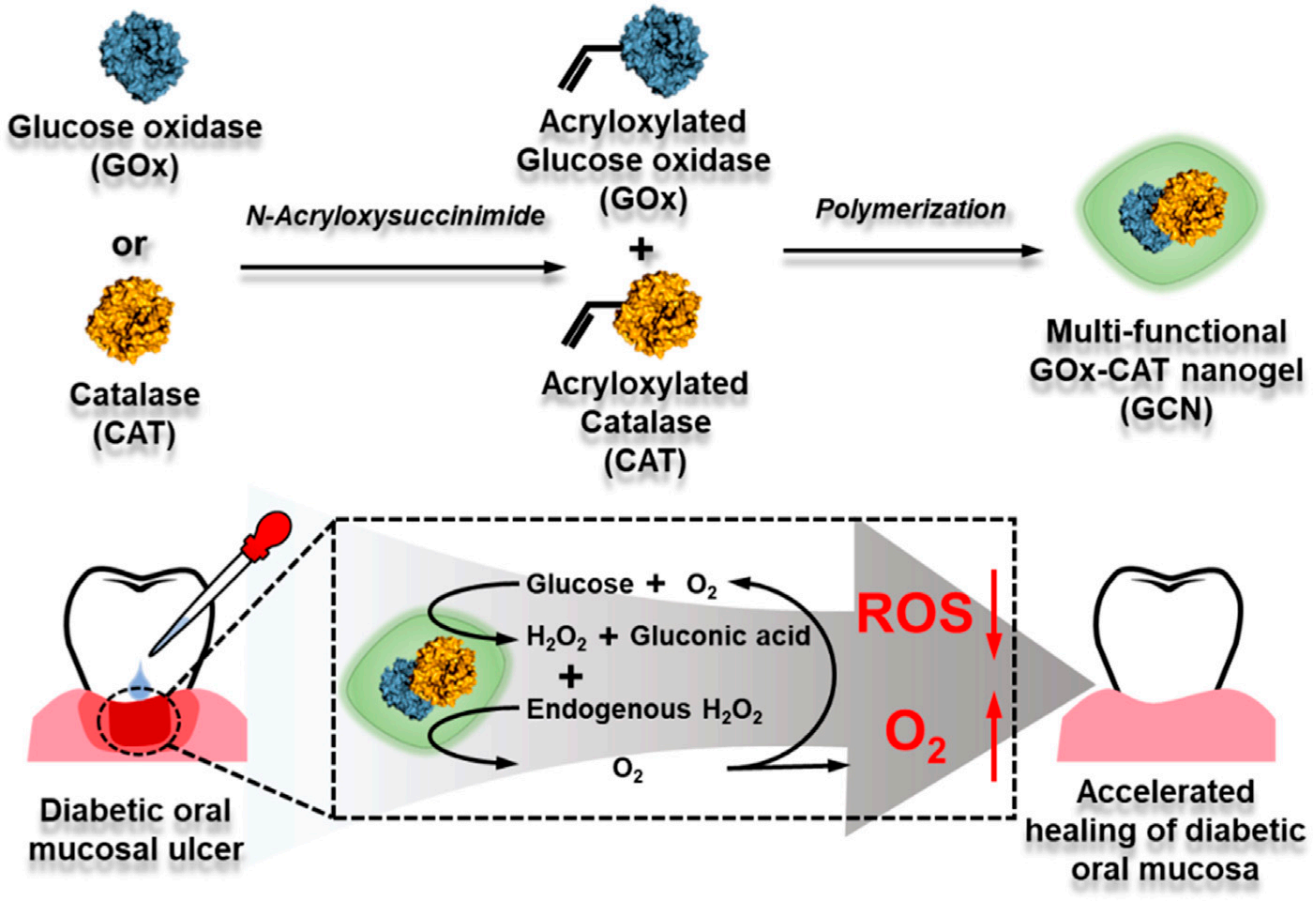
Synthetic process of GOx-CAT nanogel (GCN) and mechanism diagram of accelerating diabetic oral mucosa ulcer healing.

Considering that diabetic oral mucosa ulcers and diabetic wounds have similar characteristics, such as high-glucose exposure, high ROS, and hypoxia, in this work, a novel approach is reported based on a multi-functional GOx-CAT nanogel (GCN) system for the treatment of diabetic oral mucosa ulcers (as shown in Scheme 1). This nanogel was prepared by covalently polymerizing the natural enzyme GOx with the natural enzyme CAT. Based on the close spatial distance between GOx and CAT, GOx catalyzes the oxidation of glucose into gluconic acid and H_2_O_2_, which is transformed into oxygen (O_2_), catalyzed by CAT. In addition, the endogenous ulcer H_2_O_2_ can also be rapidly catalyzed into O_2_. *In vitro* experiments have been conducted to evaluate the GCN system in terms of cascade catalytic activity, oxygen supply effect, biocompatibility, antioxidant effect, and gingival fibroblast migration. *In vivo* ulcer healing of GCN has also been evaluated using an oral gingival mucosa model of diabetic SD rats. The experimental results suggest that this nanogel with anti-oxidation, oxygen supply, migration promotion, angiogenesis, and biocompatibility could be a promising strategy for accelerating diabetic oral mucosa ulcer healing.

## 2 Materials and methods

### 2.1 Materials

GOx nanogel (GN) and GOx-CAT nanogel (GCN) were synthetized in our previous study(Luo et al., 2021b; Fan et al., 2022). Trypsin-EDTA (ethylenediaminetetraacetic acid) was obtained from Beyotime Biotechnology. Dimethyl sulfoxide (DMSO) was supplied by Sinopharm Chemical Reagent Co. Ltd. Phosphate buffered saline (PBS) and bovine serum albumin (BSA) were supplied by Solarbio Technology Co., Ltd. 3-(4,5-dimethyl-2-thiazolyl)-2,5-diphenyl-2-H-tetrazolium bromide (MTT), penicillin-streptomycin, hematoxylin eosin (H&E staining kit), calcein-AM/PI double stain kit, and antifade mounting medium with DAPI (4ʹ,6-diamidino-2-phenylindole) were obtained from Yeasen Biotechnology Co. Ltd. Anti-fluorescence quenching tablet seal (including DAPI) was obtained from Shandong Sparkjade Biotechnology Co., Ltd. Fetal bovine serum (FBS) was obtained from Thermo Fisher Scientific Co., Ltd. A blood glucose meter was supplied by Sinocare Inc. A JPB-607A portable dissolved oxygen tester and a PHS-3C pH meter were supplied by Shanghai INESA Scientific Instrument Co., Ltd. The instruments used in the experiments were all from the Core Facility of Biomedical Sciences, Xiamen University.

### 2.2 Characterization of GCN

Transmission electron microscopy (TEM) was used to observe GCN. 1 mg GCN was dissolved in 50 μL double distilled water (dd water) and added dropwise into 950 μL double distilled water while stirring. 10 μL of the sample was dropped onto 400 mesh copper wire and left to adsorb for 10 min. Then, the copper wires with samples were used for TEM measurements. The size and zeta potential of the GCN were investigated by dynamic light scattering (DLS) measurements.

In order to investigate the catalytic activity of GOx, the pH value of the system was tested. A glucose solution (3 mg/mL) was used as a control. Other groups included GN solution (20 μg/mL), GCN solution (20 μg/mL), glucose + GN solution, and glucose + GCN solution. Different solutions were placed in an incubator at 37°C and detected at different time points with a pH meter.

In order to evaluate the catalytic activity of CAT and the cascade catalytic activity of GCN, changes in the dissolved oxygen (DO) level of the system were measured. A glucose solution (3 mg/mL) was used as a control. Other groups included GN solution (20 μg/mL), GCN solution (20 μg/mL), glucose + GN solution, and glucose + GCN solution. Different solutions were placed in an incubator at 37°C and detected at different time points with a portable dissolved oxygen tester.

In order to evaluate the cascade catalytic activity of GCN in a wound mimic environment, the DO value of the system was tested. A glucose (3 mg/mL) + H_2_O_2_ (300 μM) solution was used as a control. Other groups included a glucose + GN (20 μg/mL) solution and a glucose + GCN (20 μg/mL) solution.

### 2.3 Cell culture

Human gingival fibroblasts (HGF-1 and HGF primary generation) were kindly supplied by Qingqi (Shanghai) Biotechnology Development Co., Ltd. HGF-1 were incubated in DMEM (Gibco) containing 10% FBS and 1% penicillin-streptomycin at 37°C and 5% CO_2_.

### 2.4 Cytotoxicity assay

HGF-1 and HGF primary generation were seeded in 96-well plates at an initial cell density of 2.0×10^4^ cells per well and incubated at 37°C and 5% CO_2_ overnight. Afterward, different concentrations of GN and GCN were incubated with the cells for 24 h. Then, the medium was replaced with MTT and incubated for 4 h. Finally, cell viability was quantified using an absorbance microplate reader (CMAX PLUS, Molecular Devices, United States) to analyze the UV absorption at 490 nm.

### 2.5 Live/dead staining assay

A calcein-AM/PI double stain kit was used for live/dead staining assay of HGF-1. In brief, HGF-1 was plated at a density of 5×10^4^ per well into 24-well plates overnight. Then, the cells were incubated with 100 μm hydrogen peroxide (H_2_O_2_) for 12 h. Afterward, 5 μg/ml GN and GCN were incubated with the cells for 24 h. After fully washing the cells with 1 × assay buffer, the cells were incubated with 1 mM calcein-AM solution and 0.75 mM PI solution for 15 min. The living cells (yellow-green fluorescence) and dead cells (red fluorescence) were observed with a fluorescence microscope.

### 2.6 Scratch wound-healing assay

HGF-1 was seeded on 6-well plates at a density of 5×10^5^ per well. After 24 h, cells filled every well. Then, the cells were incubated with 400 μm H_2_O_2_ for 1 h to induce intracellular oxidative stress. A micro-injury was created using sterile 10 μl pipette tips and every well was washed three times with PBS. Then, the attached cells were treated with 5 μg/ml GN and GCN and the control group was treated with serum-free medium. The cells were photographed at 0 and 12 h after wounding using an inverted fluorescence microscope. The images were quantified by ImageJ software to evaluate the migration rate.

### 2.7 Intracellular ROS (H_2_O_2_) depletion and O_2_ evaluation assay

For the ROS depletion assay, HGF-1 cells were seeded in 24-well plates overnight and incubated with 100 μm H_2_O_2_ for 12 h. After treatment with FBS-free DMEM, GN, and GCN (5 μg/ml in FBS-free DMEM) for 24 h, the cells were incubated with DCFH-DA (10 μM in FBS-free DMEM) for 20 min. Then, the fluorescence of the cells was photographed using an inverted fluorescence microscope (Dmi8, Leica, Germany) and quantified by ImageJ software.

For intracellular O_2_ evaluation, HGF-1 cells were seeded in 24-well plates overnight and incubated with 100 μm H_2_O_2_ for 12 h. After treatment with FBS-free DMEM, GN, and GCN (5 μg/ml in FBS-free DMEM) for 24 h, the cells were incubated with [Ru(dpp)_3_]Cl_2_ (10 μg/ml) for 12 h and rinsed three times with PBS to remove free [Ru(dpp)_3_]Cl_2_. Then, the fluorescence of the cells was photographed using an inverted fluorescence microscope (Dmi8, Leica, Germany) and quantified by ImageJ software.

### 2.8 Animals and diabetic models

Sprague-Dawley (SD) rats (180–220 g) were obtained from Xiamen University Laboratory Animal Center. All animal experiments conformed to the guidelines of Xiamen University Laboratory Animal Center.

For the establishment of the type 1 diabetes model, SD rats were given a single intraperitoneal injection of streptozotocin (STZ) (60 mg/kg dissolved in sterile citrate buffer (pH 4.2–4.5); Yeasen). The next day, the blood glucose level was measured using a blood glucose meter and the unqualified rats were supplemented with STZ (30 mg/ml). Seven days later, the blood glucose level of rats was measured again and rats with plasma glucose levels higher than 16.7 mmol/L were considered diabetic. Animals were maintained in a diabetic state through the experiment.

### 2.9 Therapeutic effect of GCN on gingival mucosa ulcers

SD rats were anesthetized with 10% chloral hydrate and filter papers measuring 3 mm in length and width soaked with 80% acetic acid solution were placed in the rat gingival mucosa and left for 1 min. The next day, the above steps were repeated. On the third day, the rats were anesthetized and 20 μl aliquots of 500 μg/ml GN and GCN were applied to the ulcer sites. Three rats in each group and the control group were treated with normal saline. The mucosa ulcers were photographed with a camera every other day.

### 2.10 Histological analysis

The SD rats were euthanized after 14 days of treatment. Tissue was taken and soaked in 15% and 30% sucrose solutions for 24 h. Thereafter, frozen tissue sections with a thickness of 7 μm were generated using a freeze slicer (CM 1900, Leica, Germany). Then, the sections were stained with H&E staining for observation and analysis under a microscope.

### 2.11 Immunofluorescence staining analysis

Frozen slices of dehydrated skin tissues were generated for immunofluorescence staining. Then, the slices were fixed using ice-cold acetone and rinsed several times in PBS. After slices were permeabilized and blocked with 0.2% Triton X-100% and 5% Bovine Serum Albumin (BSA), immunofluorescence staining of TNF-α and CD31 was carried out at day 14 to assess the anti-inflammatory and angiogenesis effects.

### 2.12 Statistical analysis

All experimental data and plots were calculated using Graphpad Prism 8.0. All data were presented as the mean ± standard deviation (SD), and the difference between two group was determined using a Student’s *t*-test. A *p*-value below 0.05 was considered to be a statistically significant difference and *p < 0.05, **p < 0.01, and ***p < 0.001 are utilized for the indicated group.

## 3 Results

### 3.1 Enzymatic reaction and cascade catalytic ability of GCN

**FIGURE 1:**
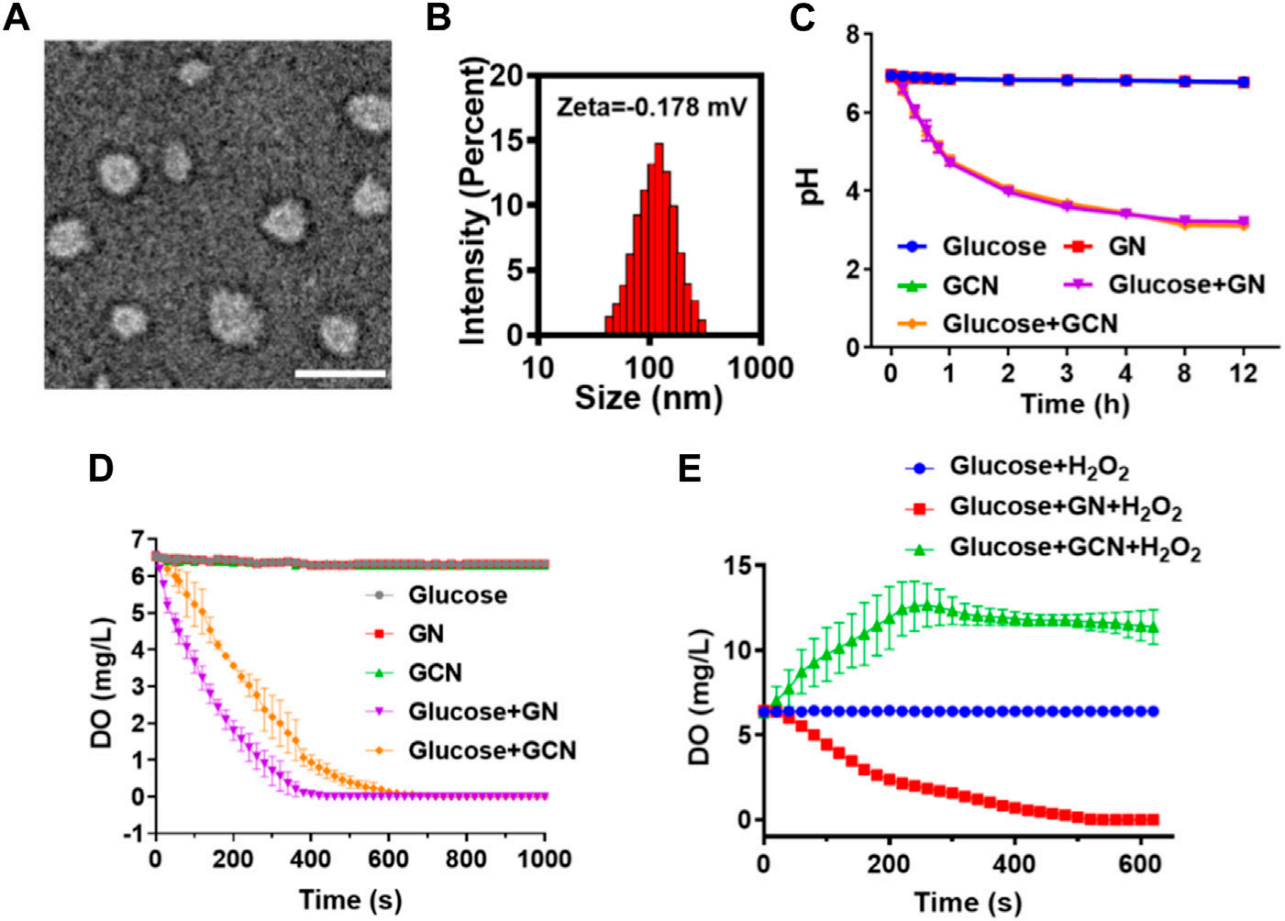
Characterization of GCN. **(A)** Representative TEM images of GCN (scale bar = 100 nm). **(B)** Particle size and zeta potential of GCN. **(C)** pH value changes of different solutions to evaluate the catalytic activity of GOx. **(D)** Dissolved oxygen (DO) value changes of different solutions to evaluate the catalytic activity of CAT and the cascade catalytic activity of GCN. **(E)** DO value changes of different solutions under a simulated diabetic oral ulcer environment.

GOx-CAT nanogels (GCN), formed by the polymerization of glucose oxidase (GOx) and catalase (CAT), were synthesized in previous study and the synthesis process is shown in Scheme 1. Firstly, morphological analysis of GCN was performed. As shown in Figure 1A, the morphology of GCN was captured by TEM imaging, which showed the uniform circular shape of GCN, with an average size of approximately 80 nm. The DLS results showed that the average size of GCN was approximately 123 nm, which was due to the water swelling ability of the nanogel. The zeta potential of the GCN was approximately −0.178 mV, which indicated that GCN had almost no charge (Figure 1B). In addition to the uniform morphology, the catalysis of GCN is key to curing ulcers. Firstly, the pH changes of the solution were detected to evaluate the catalytic ability of GOx, which is part of the GCN. The mechanism is as follows: as GOx catalyzes glucose to produce gluconic acid, the pH value of the system is lowered. As shown in Figure 1C, GOx nanogel (GN) can effectively catalyze glucose to produce gluconic acid as the pH of the system drops from 6.95 to 3.21 within 12 h. Moreover, GCN exhibited a similar catalytic ability, with the pH of the system dropping from 6.94 to 3.11 within 12 h, indicating that the polymerization of GOx with CAT did not affect the catalytic activity of GOx. The pH of the system in other groups always remained at approximately 6.8. These results verified that the GOx had excellent catalytic ability, which was not affected by polymerization. Secondly, the dissolved oxygen (DO) changes in solution were detected to evaluate the catalytic ability of CAT and the cascade catalytic activity of GCN. The mechanism is as follows: GCN is able to catalyze glucose and produce additional oxygen in a cascade reaction due to the close spatial distance between GOx and CAT. As shown in Figure 1D, when GN catalyzed glucose, the DO of the system gradually decreased from 6.57 to 0 within 440 s. This is because the catalytic reaction of GOx with glucose requires oxygen. However, when GCN catalyzed glucose, the DO of the system declined more slowly, with the DO decreasing from 6.57 to 0 within 720 s. This is because the H_2_O_2_, produced by the catalytic reaction of GOx and glucose was then catalyzed by CAT to produce additional oxygen. These results proved that CAT had excellent catalytic ability and GCN had excellent cascade catalytic ability. Last but not least, DO changes in solution were detected to evaluate the oxygen supply ability of GCN under a simulated diabetic ulcer environment, i.e., at high blood glucose levels and with oxidative stress. As shown in Figure 1E, the DO of the system decreased from 6.43 to 0 when GN was placed into the solution with glucose and H_2_O_2_, which indicated that GN did not catalyze H_2_O_2_. However, the DO of the system containing GCN increased from 6.33 to 12.667 and then stayed at approximately 11.4, which proved that GCN had excellent catalytic ability and oxygen supply ability under a simulated diabetic ulcer environment. To sum up the above results, GCN, with excellent enzymatic activity and cascade catalytic ability, can provide additional oxygen for oral ulcers under conditions of high blood glucose levels and oxidative stress.

### 3.2 *In vitro* cytotoxicity of GCN

**FIGURE 2:**
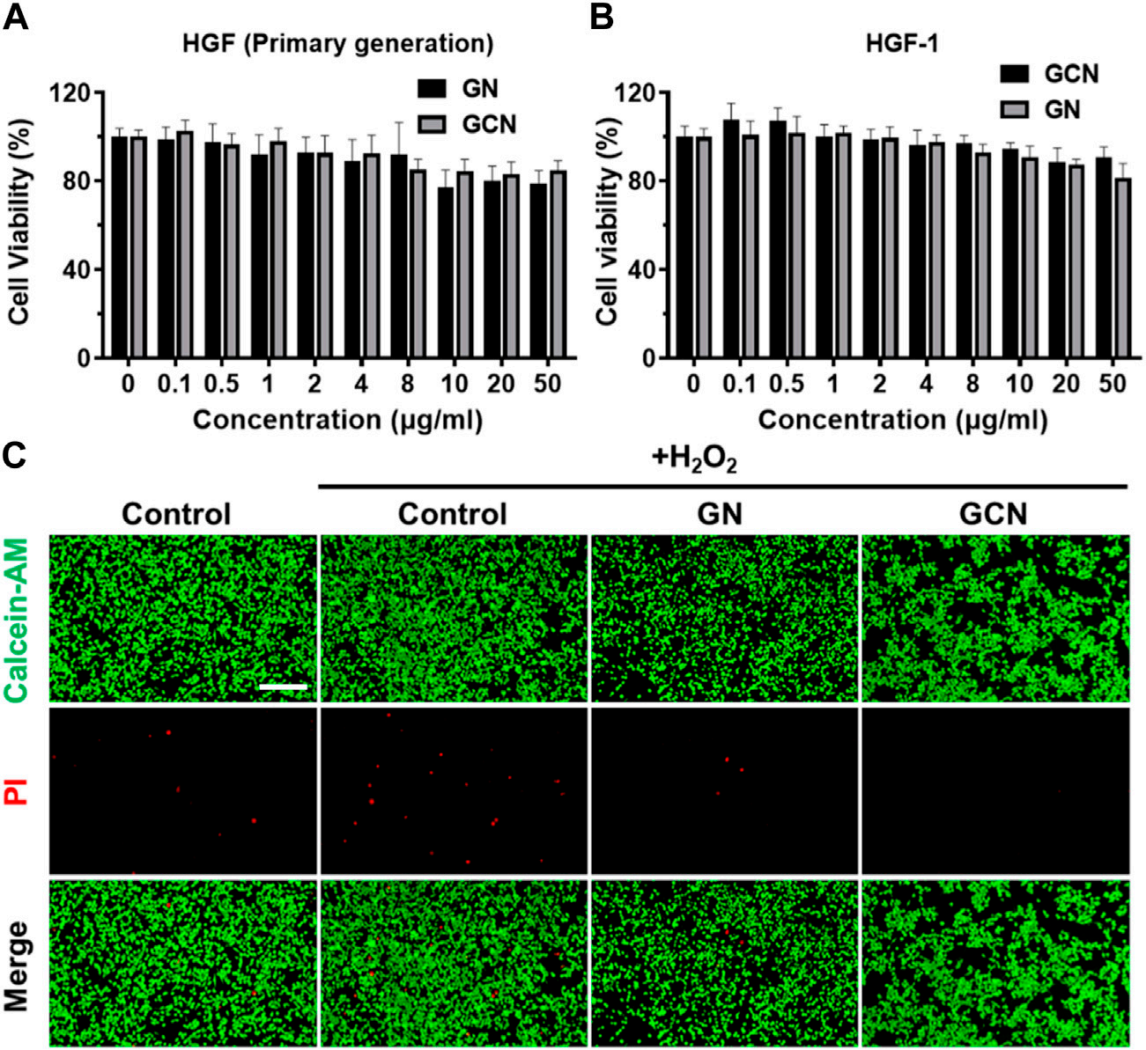
Cytotoxicity of GCN. **(A)** Cell viability of primary human gingival fibroblasts treated with GN and GCN determined by MTT assays. **(B)** Cell viability of HGF-1 treated with GN and GCN determined by MTT assays. **(C)** Live/dead staining of HGF-1 with different treatments (green fluorescence indicates live cells; red fluorescence indicates dead cells). Scale bar: 200 μm.

After evaluating the cascade catalytic ability of GCN, it is important to evaluate the effects of GCN on cells. First, the cytotoxicity of human gingival fibroblasts (HGF) was tested with an MTT assay. As shown in Figures 2A, B, an MTT assay of human gingival fibroblasts treated with GN and GCN was conducted. The results implied that low concentrations of GCN were capable of promoting cell viability. The cell viability decreased with increasing concentration, which is attributed to the accumulation of a gluconic acid byproduct produced by GOx. Nevertheless, all rates were more than 80%, indicating that GCN had no serious impact on the normal activity of cells. Simultaneously, live/dead staining was employed to examine the viability of HGF-1 cells damaged by oxidative stress after distinct treatments. As shown in Figure 2C, when HGF-1 were damaged by oxidative stress, more cells died, but this result was reversed after treatment with GCN, which could clear ROS and release oxygen to ensure the normal vitality of cells. In summary, GCN has good biocompatibility with both primary HGF and HGF-1 and can reduce the negative effects of oxidative stress damage on cell viability by clearing ROS and providing oxygen.

### 3.3 Intracellular ROS depletion, O_2_ generation, and cell migration promotion of GCN

**FIGURE 3:**
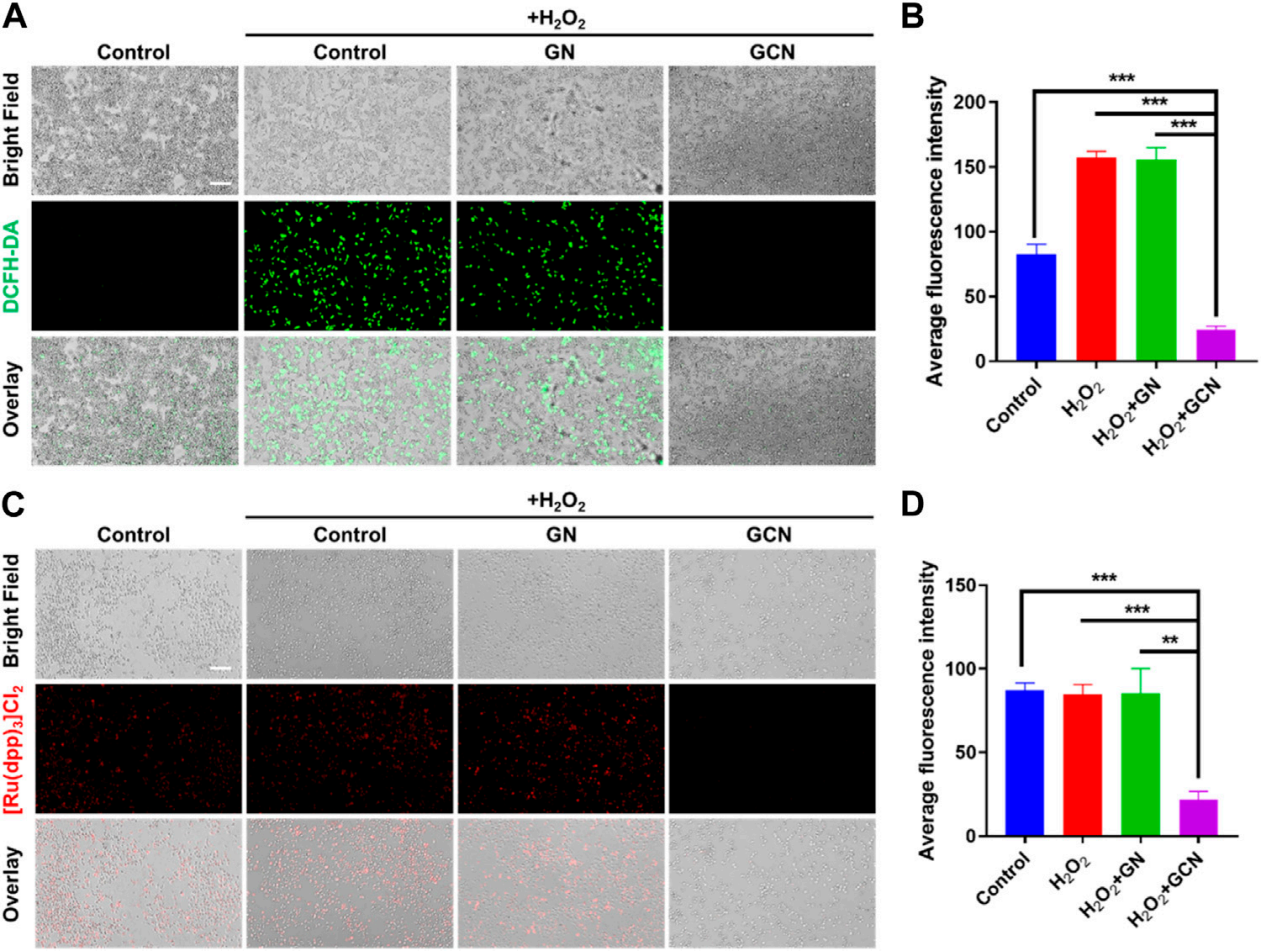
ROS scavenging and O_2_ generation ability of GCN in HGF-1 damaged by oxidative stress. **(A)** Representative fluorescent images of HGF-1 under different treatments obtained using a DCFH-DA probe. Scale bar: 200 μm. **(B)** Quantitative intracellular ROS depletion analyzed by counting the fluorescent intensity of HGF-1. **(C)** Representative fluorescent images of HGF-1 under different treatments obtained using a [Ru(dpp)_3_]Cl_2_ probe. Scale bar: 200 μm. **(D)** Quantitative intracellular O_2_ generation analyzed by counting the fluorescent intensity of HGF-1. Error bars represent the mean ± s.d. (*n* = 3, ***p < 0.001, **p < 0.01, *p < 0.05).

The above results demonstrated that GCN showed excellent cascade catalytic activity *in vitro*. Therefore, it is imperative to explore the intracellular effects of GCN. The increased production of ROS (H_2_O_2_) results in the oxidative stress of ulcers, which impedes the process of healing. We speculate that GCN could scavenge ROS and supply O_2_ to mitigate the oxidative and hypoxic microenvironment due to its CAT activity. A ROS probe (DCFH-DA) was applied to detect the intracellular ROS level in HGF-1 cells damaged by oxidative stress after treatment with GN or GCN separately. As shown in Figures 3A, B, the average fluorescence intensity of GCN-treated HGF-1 (24.65 AU) was evidently lower than that of the other three groups, which presented values of 82.60 AU, 157.08 AU, and 155.63 AU (*p* < 0.001). This phenomenon verified that GCN possessed excellent ability for intracellular ROS depletion, while GN without CAT activity did not.

Moreover, an oxygen indicator ([Ru(dpp)_3_]Cl_2_) was used to detect the O_2_ generation ability of HGF-1 cells co-incubated with H_2_O_2_. As shown in Figures 3C, D, the fluorescence of [Ru(dpp)_3_]Cl_2_ in GCN-treated HGF-1 was significantly quenched. The average fluorescence intensity in GCN-treated HGF-1 was 21.91 AU which was significantly weaker than that of the other three groups (*p* < 0.01), which presented values of 87.22 AU, 84.53 AU and 85.27 AU. This result confirmed that GCN could cascade catalyze both glucose and endogenous H_2_O_2_ in oxidative stress-damaged cells to produce oxygen.

**FIGURE 4:**
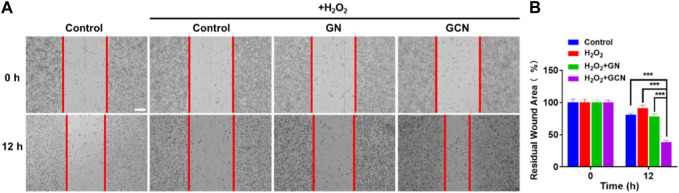
GCN promoted cell migration of HGF-1 cells damaged by oxidative stress. **(A)** Representative cell migration images of HGF-1 under different treatments. Scale bar: 200 μm. **(B)** Residual wound area analyzed by ImageJ software. Error bars represent the mean ± s.d. (*n* = 3, ***p < 0.001, **p < 0.01, *p < 0.05).

Generally, fibroblasts contribute to tissue formation and re-epithelialization during the proliferation phase of the wound healing process. Therefore, given that hypoxic and oxidative stress conditions would impair the life activities of cells in the process of wound healing, we selected HGF-1 pretreated with H_2_O_2_ as model cells to explore the influence of GCN on cell migration. As shown in Figures 4A, B, the residual wound area of HGF-1 treated with GCN was 38.75% within 12 h. Compared with the other three groups (control 80.90%; H_2_O_2_ 90.89%; GN 78.3%), GCN showed excellent ability to promote cell migration (*p* < 0.001), which proved that GCN could accelerate the process of wound healing by promoting cells migration.

In summary, GCN can alleviate intracellular ROS damage and produce oxygen to provide energy for cell activities such as cell migration via the cascade catalytic reaction.

### 3.4 GCN accelerates healing of diabetic gingival ulcers *in vivo*

**FIGURE 5:**
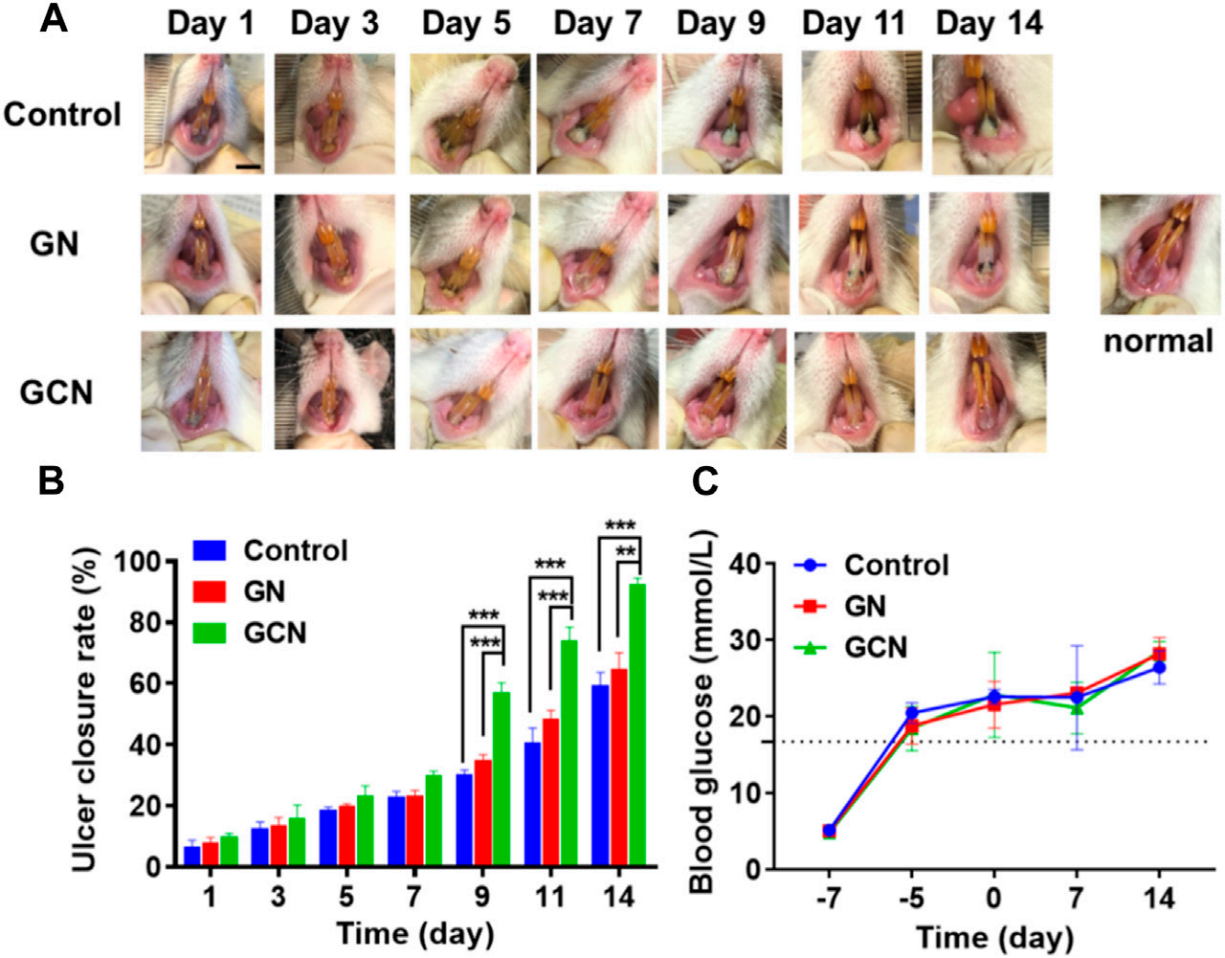
GCN-promoted healing of diabetic oral ulcers. **(A)** Representative photographs illustrating the healing of gingival ulcers over time after different treatments. Scale bar: 0.5 cm. **(B)** Change in the ulcer closure rate over time after treatment. **(C)** Change in the blood glucose level of diabetic SD rats throughout the entirety of the animal experiments. Error bars represent the mean ± s.d. (*n* = 3, ***p < 0.001, **p < 0.01, *p < 0.05).

In order to gain insight into the therapeutic effect of GCN toward diabetic oral mucosa ulcers, we established a gingival mucosa ulcer model in diabetic SD rats. As shown in Figure 5C, we continuously monitored the blood glucose levels of the tail vein over 16.7 mmol/L to guarantee that the SD rats were in a hyperglycemic state. We also measured the change in the blood glucose level of diabetic SD rats during the process of gingival ulcer healing, the result of which indicated that the gingival ulcers are simultaneously in a hyperglycemic microenvironment.

As shown in the digital photographs of oral ulcers (Figure 5A), the gingival mucosa of the GCN group became flatter than the other two groups and significant swelling or suppuration did not appear after 14 days of treatment. Furthermore, as shown in Figure 5B, the ulcer closure rate of the GCN group was 82.69% at day 14 (*p* < 0.01), which was significantly higher than the other two groups (GN group, 56.66%; control group, 52.81%). This result illustrated that GCN can accelerate the healing of diabetic gingival ulcers compared with the other two groups.

In summary, GCN with ROS scavenging and oxygen-producing activities can improve the hypoxia, high oxidative stress, and high glucose microenvironment of diabetic gingival ulcers to accelerate the healing process.

**FIGURE 6:**
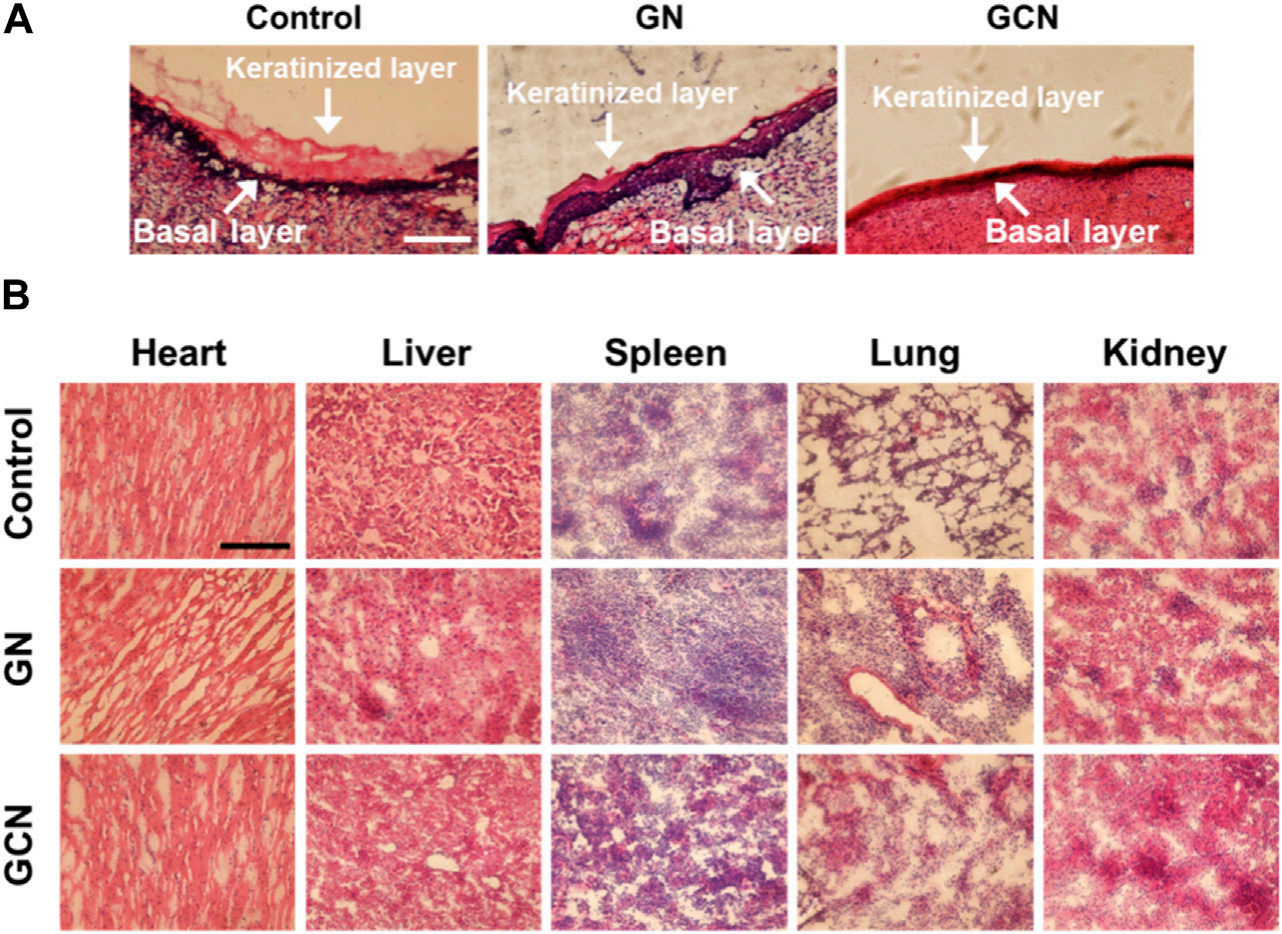
**(A)** Histological analysis by H&E staining of the gingival ulcer tissue in SD rats undergoing different treatments for 14 days. Scale bar: 100 μm. **(B)** Histomorphometric analysis of H&E staining of organs undergoing different treatments after 14 days.

Furthermore, detailed histomorphological research using hematoxylin and eosin (H&E) staining was performed to investigate the therapeutic effect on gingival ulcerous tissue. As shown in Figure 6A, in the control and GN groups, the gingival mucosa ulcers were significantly swollen, and cells between the keratinized layer and the basal layer were loosely arranged. However, in the GCN group, the keratinized layer of the epidermis was closely adhered to the basal layer and the cells were closely arranged without obvious swelling. Moreover, the number of new fibroblasts increased in the gingival tissue of the GCN group. These results confirmed that GCN accelerated diabetic gingival ulcer healing.

To further study the biocompatibility of GCN, we compared the H&E staining of the major organs (heart, liver, spleen, lung, and kidney) of rats undergoing different treatments. As shown in Figure 6B, compared to the control group, the tissue section of the GCN group did not show apparent differences, indicating that GCN had good biocompatibility and no significant biotoxicity *in vivo*.

### 3.5 Anti-inflammatory and angiogenic effects of GCN *in vivo*

**FIGURE 7:**
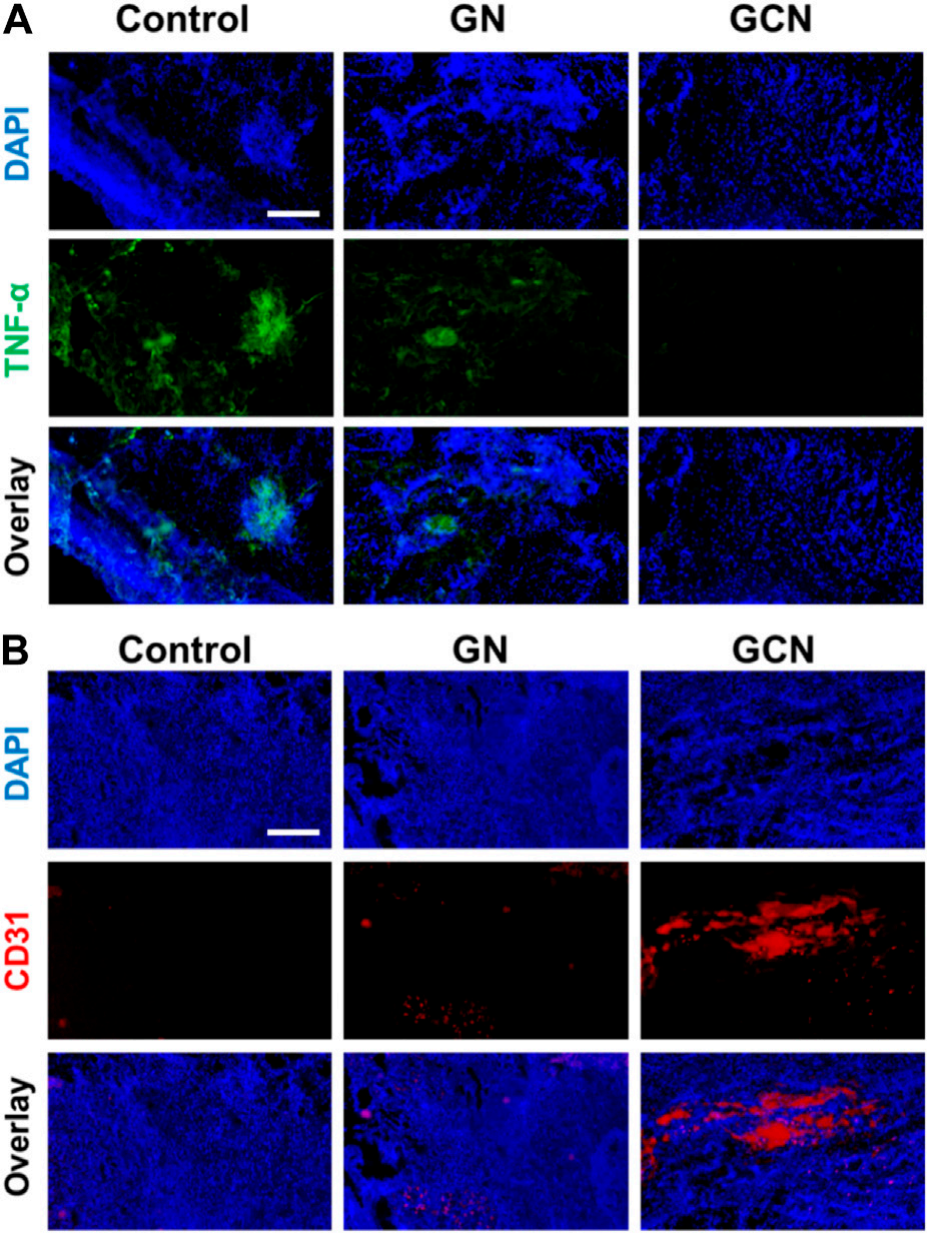
**(A)** Representative fluorescence images of TNF-α in gingival ulcer tissues. Scale bar: 100 μm. **(B)** Representative fluorescence images of CD31 in gingival ulcer tissues. Scale bar: 100 μm.

Considering that the inflammatory cytokine is a severe resistance to the healing of ulcers, TNF-α immunostaining was conducted to investigate the anti-inflammatory effect of GCN. As shown in Figure 7A, the fluorescence of the GCN group was weaker than that of the other groups, demonstrating that GCN had an admirable anti-inflammatory effect. Meanwhile, we also determined the angiogenesis of tissue using CD31 immunostaining. As shown in Figure 7B, there was plentiful red fluorescence of the GCN group, which was stronger than that of other two groups. This verified that GCN could facilitate the growth of blood vessels, which provided stimulative factors to accelerate the healing of gingival ulcers. In general, GCN with ROS depletion, O_2_ generation, and anti-inflammatory and angiogenic activities could significantly accelerate the process of gingival ulcer healing.

## 4 Discussion

Chronic wounds such as diabetic wounds are more difficult to heal because of complexities in wound microenvironment, such as hyperglycemia, hypoxia, high oxidative stress, and bacterial breeding. Diabetic oral mucosa ulcers also face these issues (Obata et al., 2021; Hamie et al., 2022). Therefore, effective wound healing agents are desperately needed. As an important energy source in the wound site, oxygen was reported to effectively promote the wound healing activities of cells (Chen et al., 2020). Recently, Liu et al. (2019) designed a responsive porous microcarrier loaded with hemoglobin for oxygen delivery to the wound. Although this trial promoted wound healing, continuous oxygen supply still faces great challenges. Therefore, we aimed to design a sustainable oxygen supply system. A GCN was designed to use endogenous H_2_O_2_ and glucose at the ulcer to achieve a cascade reaction, resulting in the continuous supply of oxygen.

In recent years, enzymes and nanozymes with enzyme-like activities have been widely reported to accelerate wound healing. For example, Tu et al. (2022) designed a hydrogel loaded with manganese dioxide, which can catalyze endogenous H_2_O_2_ to produce hydroxyl radicals on the wound surface, achieving antibacterial effects. Wang et al. (2020) designed a multifunctional hydrogel loaded with manganese dioxide to catalyze endogenous H_2_O_2_ to O_2_. However, the metal or metal oxide enzyme substances used in these studies do not guarantee a continuous catalytic efficiency or the elimination of byproducts (acid, metal ions, free radicals, etc.). Therefore, we chose a natural enzyme copolymer, which not only exhibits superior biocompatibility, but also achieves a fast cascade reaction because of close spatial connections. In addition, due to the micro efficiency of the enzyme, endogenous glucose and H_2_O_2_ at the wound site were continuously utilized by GCN to provide oxygen and relieve oxidative stress. In diabetic oral mucosa ulcers, gingival fibroblasts are in a state of oxidative stress damage, which leads to inhibition of their normal healing activities, such as cell migration. GCN can deplete excess ROS and generate oxygen to improve the cell status, thereby accelerating the closure of ulcers. These points were confirmed by both *in vivo* and *in vitro* results.

In short, the GCN developed in this report has good biocompatibility and can improve the diabetic oral mucosa ulcer environment through a glucose–H_2_O_2_ cascade catalytic system, thus promoting wound healing, which can provide a reference basis for the field of diabetic oral mucosa ulcer healing. However, it should be noted that our results verified the excellent oxygen-producing effect and antibacterial performance of the system; however, the specific mechanism by which oxygen promotes wound healing was not clarified. This is the limitation of our current research, which needs further study.

## 5 Conclusion

In summary, a nanozyme GCN system has been successfully developed, comprising two natural enzymes, GOx and CAT. The GCN possesses the functions of blood glucose regulation, anti-inflammation, and continuous oxygen supply to diabetic oral mucosa ulcers. Through the highly efficient cascade effect of GOx and CAT in its nanostructures, the GCN system can effectively regulate the topical blood glucose and ROS concentration of diabetic oral mucosa ulcers, and continuously generates oxygen to improve its hypoxic microenvironment, promoting cell migration and angiogenesis, which accelerate diabetic oral mucosa ulcer healing. Furthermore, the *in vivo* experimental results show that GCN has good biocompatibility and no obvious toxicity toward the major organs of diabetic SD rats. In short, this enzyme cascade system design might provide a novel strategy in the field of oral mucosa ulcer repair and can also be used for other diabetes-related tissue damage.

## Data Availability

The raw data supporting the conclusion of this article will be made available by the authors, without undue reservation.
